# Editorial

**Published:** 2013-03-25

**Authors:** F Popa

**Affiliations:** Carol Davila University of Medicine and PharmacyRomania

In our last Editorial (Volume V, Issue 4, 2012) we highlighted that the second edition of the International Congress “Health, Nutrition, Fitness, and Wellbeing – SANABUNAINT 2012”, took place in Falticeni, between 19-21 of October 2012, within the context of the 9th edition of the Apple Festival – “The Apples of Rădășeni”, and as an expression of the responsible preoccupation of the Falticeni Town Hall and Suceava Town Council in promoting the Public-Civic-Private Partnership, while considering the entire series of potential benefits (health, social, direct and indirect economical, environmental benefits). A first image in this Editorial showed the Opening Ceremony of the SANABUNA International Congress, which took place at the prestigious “Ion Irimescu” Art Museum, Falticeni.

Since 110 years from the birth of Master Ion Irimescu are celebrated, our welcoming “host” of the recent event “Health, Nutrition, Fitness and Welfare” – SANABUNA, which took place in Falticeni, in October 2012 (http://www.sanabuna.ro/the-second-international-congress-health-nutrition-fitness-and-wellbeing-for-central-southeast-europe-sanabuna-2012-took-place-on-the-19th-21st-october-at-the-art-museum/), our thoughts turn, with a natural emotion, to this important moment of recognizing some exemplary achievements which confirm the tough work, the commitment and the talent of the Master. The anniversary program organized by Falticeni City Council, Falticeni City Hall, “Ion Irimescu” Art Museum and “Ion Irimescu” Cultural Foundation confirm the respect of the community regarding the real values of our nation. It does not seem a coincidence that Titu Maiorescu, Founding Member of the Romanian Academy, who organized a long time ago, the well-known „Junimea” Circle meetings at Falticeni, used to say: „The art of life? Care, discreetness, moderation, basically negation and briefly abnegation”. With all his unrest in the loneliness of his workshop, Master Ion Irimescu had expressed his feelings according to the job of the local and national community, giving sense and life to the art.

I am pleased to share a significant „fresh” connection between Falticeni and Brussels, the european capital from which I have recently returned home. The words „Falticeni” and „SANABUNA” have repeatedly been pronounced at „Sfanta Ana” Castle, the office of the Belgian Diplomatic Club, on Friday the 15th of February 2013, our greeting becoming famous due to the International Congress which took place in Falticeni. This happened in the context in which: “Carol Davila” Academic Publishing House, of “Carol Davila” University of Medicine and Pharmacy in Bucharest, has been awarded The Belgian Prize of Innovation for its contribution in the organization and the successful development of SANABUNA International Congress, which took place in Falticeni (http://www.sanabuna.ro/dr-victor-lorin-purcarea-president-du-comite-national-roumain-dorganization-du-congres-international-sanabuna-a-recu-la-diplome-golden-archer-le-grand-prix-belge-de-linnovation/); the rigorous Laudatio, presented by the prestigious National School of Politic and Administrative Studies (SNSPA) Bucharest, on the occasion of granting the Doctor Honoris Causa title to Léon F. Wegnez, A.I.D.A General Secretary in Brussels, General Manager of the Belgian Diplomatic Club and co-founder of Brussels Diplomatic Gazette (http://www.crd-aida.ro/2013/02/leon-f-wegnez-doctor-honoris-causa-prestigious-snspa-bucharest-romania/), finally referred to the concept of cooperation, thanks to SANABUNA International Congress which took place in Falticeni. I am proud to say that we managed to promote the country brand in Brussels, with the help of the same professional and dedicated team in Falticeni, namely three Members of the Scientific Committee of SANABUNA International Congress, Prof. Remus Pricopie, Minister of National Education, Prof. Petru Filip, President of the Commission of Foreign Policy of the Romanian Senate, Prof. Theodor Valentin Purcărea, President of the Romanian Distribution Committee and Member of the Board of A.I.D.A. Brussels, and last, but not least, the President of the National Organizing Committee of SANABUNA International Congress, Assoc. Prof. Victor Lorin Purcărea, Director of “Carol Davila” Academic Publishing House, President of the Commission for Internal and International Policy of the Senate of “Carol Davila” University of Medicine and Pharmacy Bucharest, and Advisor to the Vice-President of the Commission of Public Health, Senate, Romanian Parliament.

Being inspired by SANABUNA International Congress, our distinguished colleague, Prof. Eliot Sorel, talked about the “Magic of Falticeni” and the uniqueness of “Ion Irimescu Museum” both in Romania and in Europe. Imbued with this state of mind from Falticeni, which we also managed to impart to Brussels, I transmitted (as Vice-President of the Commission of Public Health of the Romanian Senate, President of the International Congress SANABUNA and Honorary Member of the Romanian Distribution Committee) to the Mayor of Falticeni collegial greetings, wishing a full success for the significant anniversary event of Master Ion Irimescu. 

Highlighting the connection between SANABUNA International Congress and Brussels, I cannot stop remembering the moment in which the idea of launching SANABUNA brand shaped, on the grounds of our constant preoccupations for “Medicine and Life”. In fact, I believe that the attached images, beginning with the Brussels Diplomatic Gazette, December 2004, speak for themselves. Moreover, our discussions in the context were centered towards the legitimacy of “SANABUNA” brand. This legitimacy depends both on the vitality and the status of the brand, and on its consistency and continuity; on truly understanding the experience of the patient, while the benefits and costs for the creation of an adequate patient experience were being professionally and responsibly taken into account, while assembling tools, processes, abilities and proper measures necessary to meeting and overcoming the needs of the patients, rethinking the system of improving the healthcare system, while having the patient as the core element. All these imply a greater commitment in accepting the challenge of a creative thinking, managing the problem with regard to the quality of life, which is connected to health and the health perceived by the patient, making a progress by knowing and understanding the reciprocal confidence between the healthcare provider and the patient, while approaching the relationship between medicine (as a science and art) and life, finally giving life to the years.

 Prof. Dr. Florian Popa

**Fig. 1:**
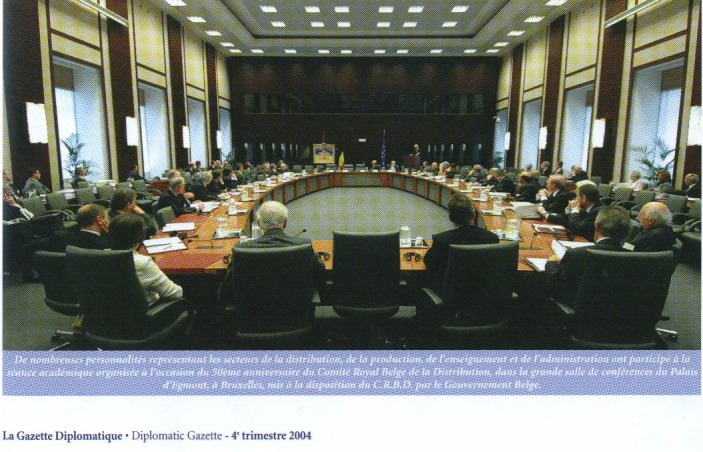
Brussels Diplomatic Gazette, December 2004, Palais d’Egmont

**Fig. 2:**
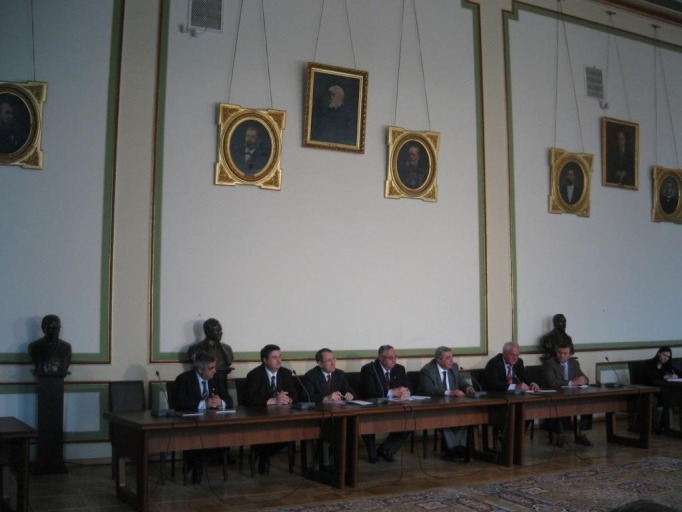
SANABUNA Conference 2009

**Fig. 3:**
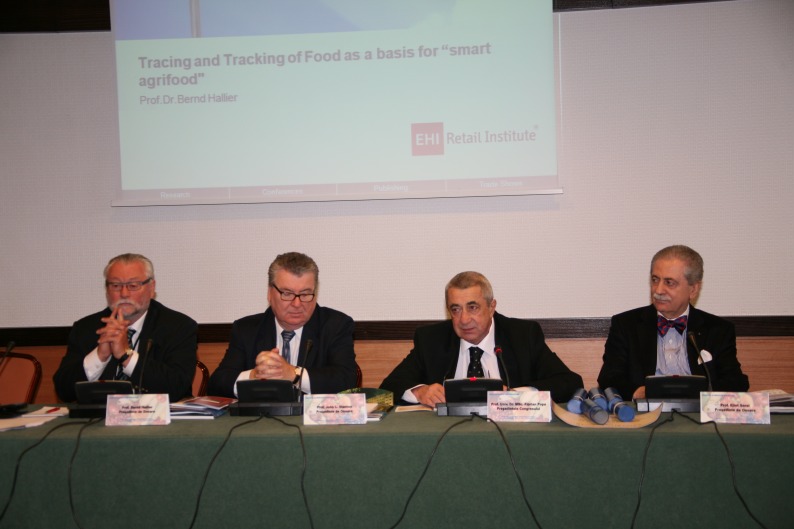
SANABUNA International Congress, 2011

**Fig. 4:**
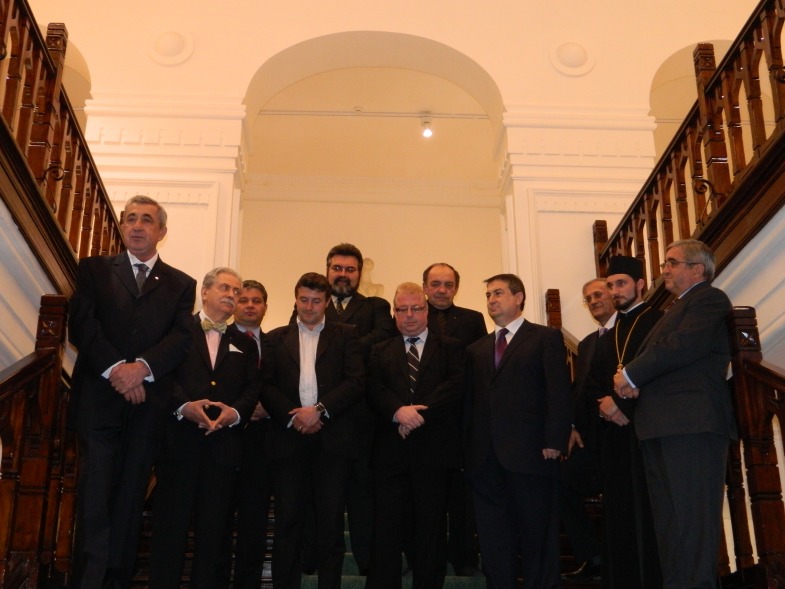
SANABUNA International Congress, 2012

**Fig. 5:**
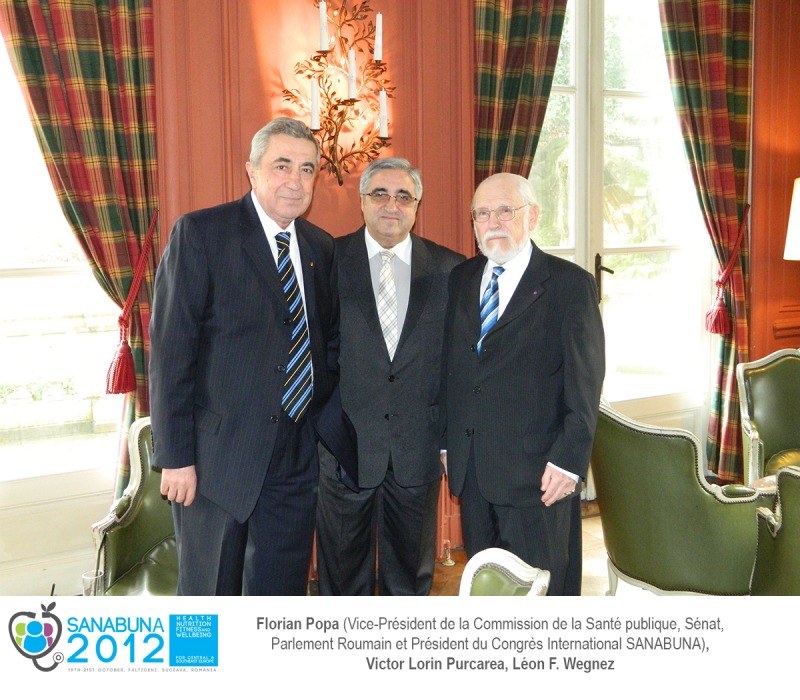
Brussels, Chateau Saint-Anne, 2013

